# Imprint cytology of osteosarcoma of the jaw: a case report

**DOI:** 10.1186/1752-1947-3-9327

**Published:** 2009-12-11

**Authors:** Luiz Antonio Guimarães Cabral, Cristina Werkman, Adriana Aigotti Haberbeck Brandão, Janete Dias Almeida

**Affiliations:** 1 Department of Biosciences and Oral Diagnosis, São José dos Campos Dental School, São Paulo State University - UNESP, São José dos Campos, São Paulo, Brazil; 2 Postgraduation Program in Oral Biopathology, São José dos Campos Dental School, São Paulo State University - UNESP, São José dos Campos, São Paulo, Brazil

## Abstract

**Introduction:**

Osteosarcomas are highly malignant bone-forming neoplasms that account for about 20% of all sarcomas. In light of their aggressive behavior, early diagnosis is crucial for determining adequate treatment. Dental professionals may be the first to detect jaw osteosarcomas in their initial stages. The aim of this case report is to draw attention to the possibility of diagnosing this tumor based on clinical, radiographical and cytological characteristics before confirmation by histology.

**Case presentation:**

A 24-year-old Afro-Brazilian man presented with swelling and pain on the left side of the mandible in the region of the third molar (tooth 38). Radiography showed a poorly delimited intraosseous lesion with radiolucent and radiopaque areas. The cytological aspects were consistent with the diagnosis of osteosarcoma, which was confirmed by biopsy.

**Conclusion:**

Imprint cytology was found to be a reliable, rapid and easy complementary examination. An early diagnosis of osteosarcoma of the jaw is fundamental to the early determination of an adequate treatment.

## Introduction

Osteosarcomas are rare malignant neoplasms of mesenchymal origin with a high rate of mortality. They are characterized by the proliferation of neoplastic cells that are able to directly form bone or osteoid tissue [1]. Excluding hematopoietic neoplasms, osteosarcomas are the most common intraosseous tumors. The tumor shows a discrete predominance in men and affects all ages from small children to the elderly. The highest prevalence of osteosarcoma is observed during the second decade of life, which coincides with the period of maximal bone growth. The tumor most commonly occurs in the long bones, mainly affecting the distal metaphysis of the femur, proximal region of the tibia and metaphysis of the humerus close to the shoulder. A second peak incidence is observed in adults above the age of 50, with the tumor involving the axial skeleton and flat bones such as vertebrae and the hip bone [1-5].

Osteogenic sarcoma of the jaw bones (JOS) corresponds to only six to seven of all osteogenic sarcomas. JOS mainly occurs in the third and fourth decades of life. However, in certain geographic regions such as Africa, JOS may affect patients at a younger age than that found in other regions of the world, with the mean age being 27.2 years [4,6].

JOS affects the maxilla and mandible at around the same frequency [4,6]. Tumors located in the mandible are more common in the posterior region of the mandibular body, whereas maxillary tumors more frequently arise from the posterior portion of the alveolar ridge, antrum, sinus floor and palate than from the zygoma or orbital ridge [1,4,7].

The main clinical manifestations of JOS are pain of variable intensity and swelling of bone and adjacent soft tissues. Tooth bulging and dislocation, lack of healing and swelling at the site of tooth extraction, trismus, hypoesthesia or paresthesia in the case of mandibular tumors, and nasal obstruction in maxillary tumors have also been reported [6,7].

Radiography reveals variable bone density depending on the amount of bone formed by the neoplasm. In some cases, the typical "sunray" appearance is observed at the periphery of the tumor. Imaging exams are important and essential for the evaluation of the tumor. Axial computed tomography is a valuable tool in evaluating bone destruction and production by the neoplasm and permits the precise definition of the extraosseous extent of the tumor and its relationship with neighboring tissues. Another important exam for the diagnosis of invasion of tumor-adjacent tissues is nuclear magnetic resonance imaging, which is extremely valuable for preoperative staging [1].

Regardless of their location, osteosarcomas are classified based on the anatomical location of the affected bone and histological pattern [8]. There is general consensus that no correlation exists between the histological type and prognosis. However, it is important to evaluate the grade of cellular atypia since some investigators have attributed a better prognosis to low-grade chondroblastic tumors in their series [7,9].

Treatment protocols for osteosarcoma include radical or conservative surgery complemented by radiotherapy and/or chemotherapy. In a multicenter study, the Brazilian Osteosarcoma Treatment Group evaluated the impact of chemotherapy and surgery on the outcome of osteosarcoma of the extremities in Brazilian patients [10].

This article reports a case of JOS diagnosed by imprint cytology.

## Case presentation

**Figure 1:**
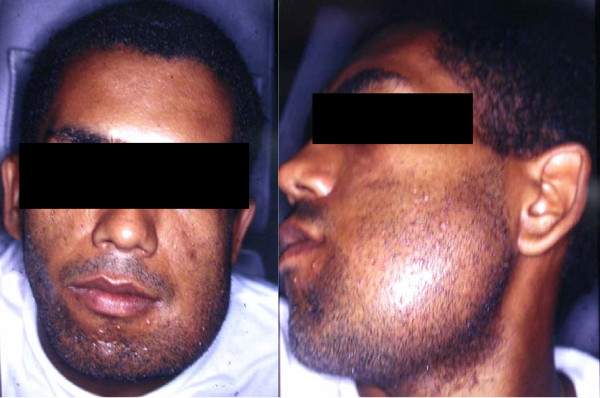
**Extraoral examination showed a swelling causing deformity of the left mandibular body**.

**Figure 2:**
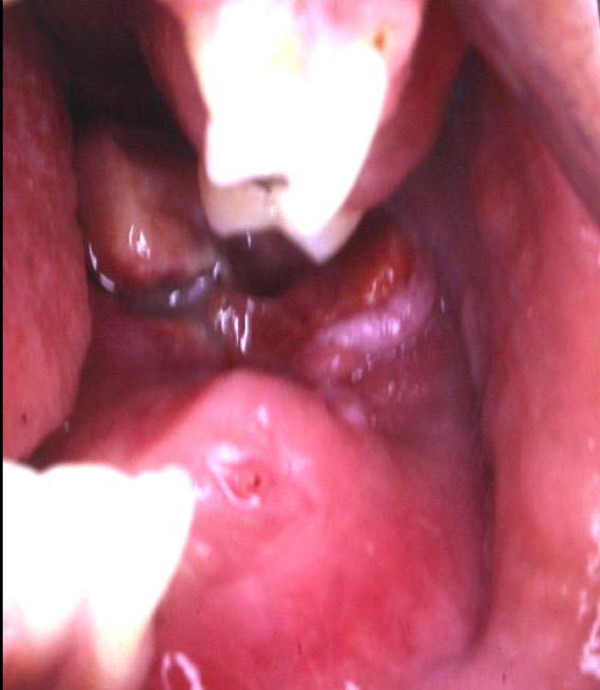
**Clinical intraoral examination showing tissue proliferation which inverted the fundus of the buccal pouch from the region of tooth 33 to the retromolar triangle**.

Our patient was a 24-year-old Afro-Brazilian man, who was married and worked as a garbage collector. He had a four-month history of pain in the region of tooth 38. Extraoral examination showed a swelling causing deformity of the left mandibular body (Figure 1), which was asymptomatic to touch and associated with paresthesia of the left hemimandible. There was no alteration in skin color or temperature. Clinical intraoral examination revealed tissue proliferation which inverted the fundus of the buccal pouch from the region of tooth 33 to the retromolar triangle (Figure 2). Tissue necrosis surrounded by a telangiectatic area was observed.

**Figure 3:**
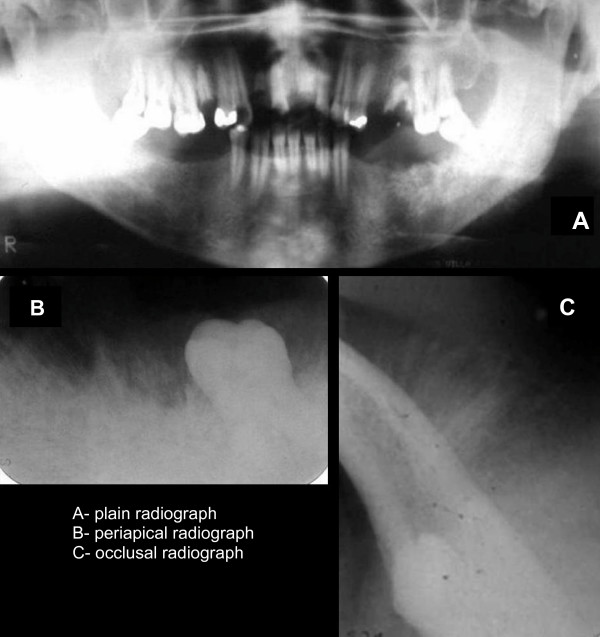
**Orthopantomography (A) and occlusal (B) and periapical (C) radiographs reveal diffuse bone destruction on the left side of the mandible, presenting dense radiopaque, mixed and completely radiolucent areas**. The periosteum showed the classical "sunray" reaction on occlusal (B) and periapical radiographs (C).

Periapical and occlusal radiography and orthopantomography revealed diffuse bone destruction on the left side of the mandible due to the presence of a lesion of variable appearance, presenting dense radiopaque, mixed and completely radiolucent areas. The lesion was extensive and poorly delimited, with the periosteum showing the classical "sunray" reaction on occlusal and periapical radiographs (Figure 3).

**Figure 4:**
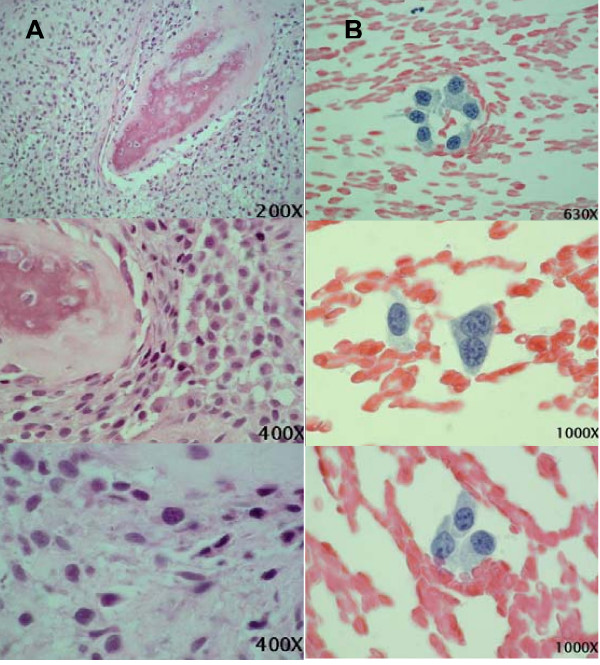
**A. Histological aspect of the biopsy material**. B. Cytological aspects of the imprint material.

An incisional biopsy was obtained from the central area of the lesion and imprint cytology was performed immediately after tissue removal. Cytological analysis revealed the presence of pleomorphic malignant cells containing well-delimited basophilic cytoplasm. The cells were oval, spindle-shaped or irregular in shape or even presented multiple prolongations. The nucleus was oval or round and voluminous and contained coarse granular chromatin and enlarged nucleoli. Nuclear membrane indentations were sometimes observed. A small amount of pale eosinophilic amorphous material compatible with osteoid substance and numerous red blood cells were also noted. The diagnosis was Papanicolaou class V, compatible with a malignant neoplasm of mesenchymal origin and with the clinical diagnosis of osteosarcoma (Figure 4B).

The anatomopathological exam was conclusive and the diagnosis was osteoblastic osteosarcoma. The tumor was characterized by the presence of solid areas containing numerous oval, round, spindle-shaped or elongated irregular pleomorphic cells, intermingled with numerous irregular fragments of mineralized bone containing various cells and abundant osteoid tissue (Figure 4A).

The patient was referred to an oncologist and a head and neck surgeon and was submitted to hemimandibulectomy, followed by 33 sessions of radiotherapy. After radiotherapy, the patient returned to the dental clinic with trismus and gingival bleeding in the region of the lower incisors and upper molars and premolars. The patient was diagnosed with pseudomembranous candidiasis in the region of the alveolar ridge, hard and soft palate and the back of the tongue. Despite treatment, the patient died six months after the diagnosis.

## Discussion

The age of the young adult patient in this case report was below that reported in the literature for JOS [6]. Clinically, the patient showed standard signs and symptoms of tissue growth that led to bone swelling and invaded adjacent soft tissues, causing pain and tooth displacement [8]. The patient also reported an onset of symptoms three to four months earlier and paresthesia, findings indicative of a relatively slow and expansive growth.

Periapical and occlusal radiography and orthopantomography, routine exams in dental care services, permitted the detection of an extensive lesion presenting variable radiographical densities. In addition, classical "sunray" reactions of the periosteum were detected on an occlusal radiograph, a feature observed in about 25% of cases.

Imprint cytology of biopsy material permitted the observation of voluminous malignant neoplastic cells containing slightly basophilic spindle-shaped or pleomorphic cytoplasm emitting one or two prolongations. The cytoplasm contained microvacuoles and large round to oval pleomorphic and hyperchromatic nuclei, which sometimes presented an indented nuclear membrane. Coarse granular chromatin and enlarged and/or multiple nucleoli were observed. In addition, portions of osteoid substance were noted, a finding important for the diagnosis. The cytological exam permitted the diagnosis of a malignant mesenchymal tumor highly suggestive of osteosarcoma within a short period of time and even before the biopsy result. In addition, the imprint method permitted the collection of cells of excellent quality for analysis and more refined visualization of cellular aspects than in biopsy material, considering that the cells suffer modifications during the process of decalcification of the material [11-14].

Cytological analysis was found to be a useful complementary exam for the preoperative diagnosis of osteosarcoma, and was important to reduce the time until referral of the patient for treatment. After diagnosis, the patient underwent surgical removal of the tumor with a safety margin, followed by chemotherapy as recommended in the literature. The prognosis depends on various individual aspects such as early diagnosis, tumor stage, surgical margins achieved, and the type of treatment instituted.

The present case demonstrates the validity and importance of less expensive examinations such as dental radiographs and cytological analysis. It must be noted, however, that these do not replace more refined techniques.

## Conclusion

Imprint cytology was found to be a reliable, rapid and easy complementary examination. An early diagnosis of JOS is fundamental for the rapid institution of adequate treatment.

## Abbreviations

JOS: Osteogenic sarcoma of the jaw bones.

## Consent

Written informed consent was obtained from the patient for publication of this case report and any accompanying images. A copy of the written consent form is available for review by the Editor-in-Chief of this journal.

## Competing interests

The authors declare that they have no competing interests.

## Authors' contributions

JDA performed the biopsy, LAGC performed the cytological imprint, and both analyzed and interpreted the clinical data of the patient. AAHB performed the cytological and histological examination. CW was a major contributor in writing the manuscript. All authors read and approved the final version of the manuscript.
